# Cancer Stem Cell Characteristics by Network Analysis of Transcriptome Data Stemness Indices in Breast Carcinoma

**DOI:** 10.1155/2020/8841622

**Published:** 2020-10-06

**Authors:** Zeng-hong Wu, You-jing Zhang, Chang-Li Jia

**Affiliations:** ^1^Department of Infectious Diseases, Union Hospital, Tongji Medical College, Huazhong University of Science and Technology, Wuhan 430022, China; ^2^Department of Otorhinolaryngology, Union Hospital, Tongji Medical College, Huazhong University of Science and Technology, Wuhan, Hubei, China; ^3^School of Public Health, Tongji Medical College, Huazhong University of Science and Technology, Wuhan 430022, China; ^4^School of Health Science, Wuhan University, Wuhan, Hubei Province 430072, China

## Abstract

**Objective:**

Breast cancer (BC) affects women all over the world. This study aimed at screening out potential biomarkers through performing an in-depth analysis of data from the previous research and database.

**Design:**

This study made full use of RNA sequencing (RNA-seq) data from cancer genomic maps (TCGA) and screened key genes related to stemness by merging WGCNA with BC mRNAsi.

**Results:**

The related mRNAsi data were downloaded, and the transcriptional levels of mRNAsi in cancers contrasted with normal samples. The results showed that there was a significantly higher mRNAsi expression in BC tissues (*P*=1.791*e* − 43). Seven modules were obtained following the investigation through cluster analysis. The turquoise module showed a relatively high positive correlation with mRNAsi at 0.79; this module was chosen as the most interesting and was used for subsequent analysis. By setting related cutoffs, 38 key genes were screened, and the coexpression of these genes was explored next. The results showed that the lowest correlation was between *CDC20* and *KIF11* (0.54), and the highest connection was between *BUB1* and *CKAP2L* (0.86). Furthermore, ten hub genes with the most nodes were sorted using a histogram. Using other databases to explore the prognosis value of key genes, the results showed that lower expression of key genes was significantly connected with longer overall survival (OS), distant metastasis-free survival (DMFS), and relapse-free survival (RFS). The immune infiltration relationship between hub genes and six kinds of basic immune cells was investigated; it was revealed that partial ones were positively or negatively related.

**Conclusion:**

This study is the first to show the important role of stemness-related genes in the prognosis of BC. However, future clinical trials are needed to confirm these results and promote the application of these key genes in prognosis evaluation.

## 1. Introduction

Breast cancer (BC) easily metastasizes to the bones and lungs, has the highest incidence rate, and is the second leading cause of death among women [1, 2]. The disease accounts for 23% of all cancer deaths, according to the World Health Organization (WHO) 2012 reports [3]. One in every three women in Asia is at the risk of BC in their lifetime [3]. Early diagnosis and intervention are particularly important. The malignancy is caused by complex inherited and environmental factors. The known hazard factors for BC include high alcohol consumption and physical inactivity. Unfortunately, few symptoms curtail early diagnosis and often lead to serious consequences if the disease is diagnosed at the advanced stage [4].

Several studies in recent years have shown that stem cell-like cell populations, which are distinct from the cancer bulk cells (bCSC), are a major factor influencing recurrence and progression of BC [5]. Breast cancer is generally divided into 5 intrinsic molecular subtypes, including luminal A, luminal B, HER-2 enriched, basal-like, and Claudin-low speaking according to the sequencing of the BC genome and transcriptome [6]. Remarkable progress has been achieved in the treatment of early breast cancer including a combination of drugs, radiation therapy, and surgery [7]; however, due to huge cytotoxicity and poor efficacy on advanced tumors, BC patients have a disappointing 5-year survival rate. The *PDE3A* gene, which is regarded as a mediator of cancer stemness, could predispose breast cancer patients to metastases [8]. Cancer stem-like properties in BC have a vital role in overcoming resistance [9]. Zaoui et al. found that breast-associated adipocytes potentiate the invasiveness of breast cancer cells [10]. The microenvironment of BC tissues has attracted much attention recently, in which oxidative stress is considered as a determining factor in the proliferation and growth of breast cancer cells [11]. Similarly, the study also reported that cancer stem cells are recognized as a key regulator of malignancy as a result of causing metastasis, relapse, and therapy resistance [12].

Microarray technology and bioinformatics analysis have been widely used to throughput and simultaneously detect thousands of genes at the genome level. A study based on various platform analysis of methylomes, transcriptomes, and transcription factor binding sites to quantify stemness and an mRNA expression-based stemness index (mRNAsi) from various cancers were obtained. Therefore, the mRNAsi data were download for our analysis [13]. The weighted gene coexpression network analysis (WGCNA) and its set of coexpressed genes were explored by making use of RNA sequencing (RNA-seq) data from cancer genomic maps [14] (TCGA). Overall, this study attempted to filter determined key genes related to stemness and select biomarkers for diagnosis.

## 2. Methods

### 2.1. Gene Information and Bioinformatics Analysis

Information on gene expression (1164 tissues, workflow type: HTSeqCounts) and clinical data (1054 cases, data format: BCR XML) was obtained from level 3 gene expression information (FPKM normalized) of the TCGA BC cohort. Also, the mRNAsi expression level we obtained before is an indicator showing the resemblance between stem cells and tumor cells and therefore can be regarded as a quantitative indicator. These samples were combined into a matrix file using a merge script in the Perl language. The clinicopathological data collected included age, stage, grade, T-stage, M-stage, N-stage, survival status, and survival duration in days. Additionally, boxplots relevant to clinical and sample data were applied to foresee expression differences of discrete variables, which were examined using the R (version 3.5.3) and R Bioconductor packages. The Ensembl database (http://asia.ensembl.org/index.html) was used to transform gene names from Ensembl IDs to corresponding gene symbols. Data matrix and data processing were made using the Perl language (*P* < 0.5). The listwise deletion technique was used to deal with missing data; the entire sample was excluded if any value was absent. The Kruskal–Wallis test was used to determine the significance of differences between subtypes by examining the connection between clinical factors and mRNAsi.

### 2.2. Identification of Differentially Expressed Genes (DEGs)

The “edgeR” R package was utilized to measure the identification of DEGs between BC and noncancerous samples. The adjusted *P* value and Benjamini and Hochberg false discovery rate method were used to correct the discovery of statistically significant genes and limitations of false positives. |log 2FC| > 1 combined with *P* value <0.05 was considered statistically significant. Other noncompliant data were not be adopted.

### 2.3. WGCNA and Module Preservation

WGCNA describes the association between genes across the entire microarray sample. The heterogeneity accuracy of bioinformatics statistics is the basis of coexpression network analysis [15], so the genes with the most differential expression were screened. Artificial threshold parameters were set to avoid information loss and filter RNA-seq information to decrease outliers because of the successive nature of coexpressed data. A weighted adjacency matrix was then constructed and transformed into a topological overlap matrix (TOM), which can evaluate the direct correlation of gene pairs and the degree of association with other genes in the dataset as well as the network connectivity. The suitable minimum gene module dimension was set for the gene tree, to merge similar genes into an independent module. A modular eigengene (ME), which can be deemed to be the main component of a modular gene expression profile, is defined as the feature expression profile within the module of interest. Module significance (MS), defined as the average GS, played a vital role in measuring relevance between the module and sample traits. Marker genes, regarded as the heart of the network architecture, are highly connected central nodes. For each gene, a module membership (MM) was determined by associating the module's gene expression profile with the ME of a particular module. The threshold for filtering key genes in the module is defined as cor. gene GS > 0.5 and cor. gene MM > 0.8.

### 2.4. KEGG and GO Enrichment Analyses

The Kyoto Encyclopedia of Genes and Genomes (KEGG) [16] is a database resource aimed at exploring key genes' functions and their biological functions. Gene ontology (GO) function analysis (biological processes (BP), cellular components (CC), and molecular functions (MF)) is an essential tool for analyzing biological process and annotate genes. The “clusterProfiler” R package was used to perform GO functional annotations and KEGG pathway enrichment analysis.

### 2.5. Protein-Protein Interaction (PPI) Network Construction

An online database (STRING; http://string-db.org) [17] was used to search for the retrieval of interacting genes and predicting the PPI network information. Analyzing the interactions and functions between DEGs may provide information about the mechanisms of generation and development of disease (PPI score > 0.4). Interestingly, the number of adjacent nodes of each gene can be calculated, based on the genes sorted by a histogram. Typically, genes with the most nodes are considered key genes.

### 2.6. Modification Species Analysis of Stemness-Related DEGs

The cBioPortal [18] is an open free asset that visualizes, analyzes, and downloads large-scale cancer genomics datasets; it was used to perform modification species analysis in stem-associated DEG in TCGA BC samples. Furthermore, various datasets could be selected for different purposes.

### 2.7. Kaplan–Meier Plotter

Kaplan–Meier plotter (http://kmplot.com/analysis/) [19] collects gene expression and prognosis data from patients across 21 cancer types, including BC. From this dataset, users can obtain the survival significance of mRNAsi expression levels. The prognosis of multigenes was explored by setting “use multiple genes” which involved OS (overall survival), PPS (postprogression survival), DMFS (distant metastasis-free survival), and RFS (relapse-free survival). Additionally, the prognosis of patients in different subgroups was analyzed by setting different parameters, including patients with different pathologies, treatment modes, and datasets.

### 2.8. TIMER Analysis

TIMER [20] is an open-access web interface used to systematically study the immune infiltration of various malignancies. The abundances of six kinds of immune cells (B cells, CD8^+^ T cells, CD4^+^ T cells, macrophages, neutrophils, and dendritic cells) were evaluated, which was assessed using our statistical methods and pathology. Hub genes with the most nodes have been obtained before. Six immune infiltrates' abundances of 10 hub genes were acquired using this immune infiltration module (*P* value <0.05).

## 3. Results

### 3.1. mRNAsi and Clinical Characteristics in BC

**Figure 1:**
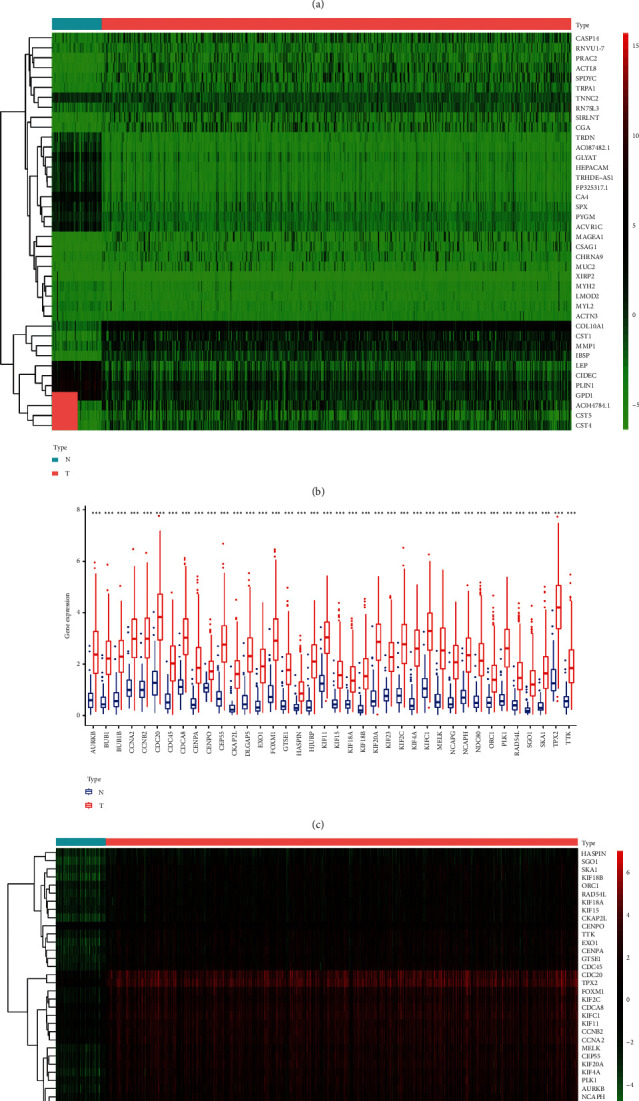
(a) The connection between clinical factors and mRNAsi expression. The results demonstrate that mRNAsi correlated significantly with the patient T and stage classifications. (b) The heatmap of top 20 upregulated and downregulated DEGs between the BC and normal samples. (c) The different expression transcriptional levels of 38 key genes in cancers and normal samples. All key genes had significantly higher expressions in BC tissues. (d) The heatmap of key genes in the turquoise module.

The mRNAsi data from Pan statistics [11] were first downloaded, and the transcriptional levels of mRNAsi in cancers and normal samples contrasted. Significantly higher mRNAsi expressions were found in BC tissues compared with normal ones (*P*=1.791*e* − 43). The connection of clinical factors and mRNAsi expressions was then explored; the results demonstrated that mRNAsi correlates significantly with the patient's T (*P* < 0.001) and stage (*P* < 0.001) classifications (Figure 1(a)). The findings above suggest that the expression of mRNAsi was especially different and may have a pivotal role in regulating BC development. Data cleansing to select differential genes was carried out as the expression level of mRNAsi in normal samples is remarkably dissimilar from that in carcinoma. Data normalization, filtering, and difference analysis were then performed to contrast BC with normal specimens. From this analysis, the heatmap of the top 20 upregulated and downregulated DEGs was shown (Figure 1(b)).

### 3.2. WGCNA Construction and Module Preservation Analysis

**Figure 2:**
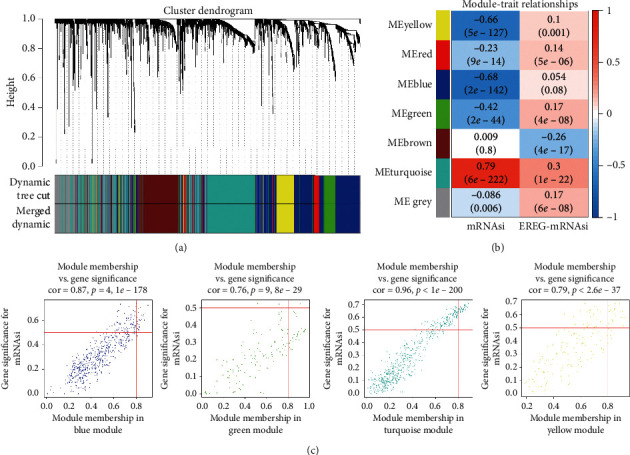
(a) Using cluster analysis, DEGs with a variance of up to 25% were placed in one module, and 7 modules were obtained for further analysis. (b) The turquoise module was most remarkably relevant to mRNAsi, with a correlation near to 0.79. The blue module showed a relatively high negative correlation with mRNAsi, with a correlation of −0.68. (c) Scatter plot of module eigengenes in the blue, green, yellow, and turquoise modules.

WGCNA was used to build a gene coexpression network to describe gene modules and genes connected with tumor stem cells. Using cluster analysis, DEGs with a variance of up to 25% were placed in one module, and 7 modules were obtained for the subsequent analysis (Figure 2(a)). The turquoise module was most remarkably relevant to mRNAsi, with a correlation near 0.79. The blue module showed a relatively high negative correlation with mRNAsi (−0.68) (Figure 2(b)). Scatter plot of module eigengenes is given in the blue, green, yellow, and turquoise modules (Figure 2(c)). Therefore, the turquoise module was selected as the most interesting module, and it was used for subsequent analysis. Thirty-eight key genes were screened including *TPX2*, *HJURP*, *PLK1*, *CDCA8*, *KIFC1*, *KIF4A*, *EXO1*, *KIF2C*, *CCNB2*, *NCAPG*, *NCAPH*, *CENPA*, *KIF20A*, *TTK*, *MELK*, *KIF23*, *RAD54L*, *KIF18B*, *BUB1*, *NDC80*, *ORC1*, *SGO1*, *BUB1B*, *CKAP2L*, *SKA1*, *CDC45*, *CDC20*, *DLGAP5*, *FOXM1*, *KIF15*, *AURKB*, *CCNA2*, *HASPIN*, *KIF18A*, *CEP55*, *CENPO*, *KIF11*, and *GTSE1*.

### 3.3. Functional Enrichment Analysis of Key Genes

**Figure 3:**
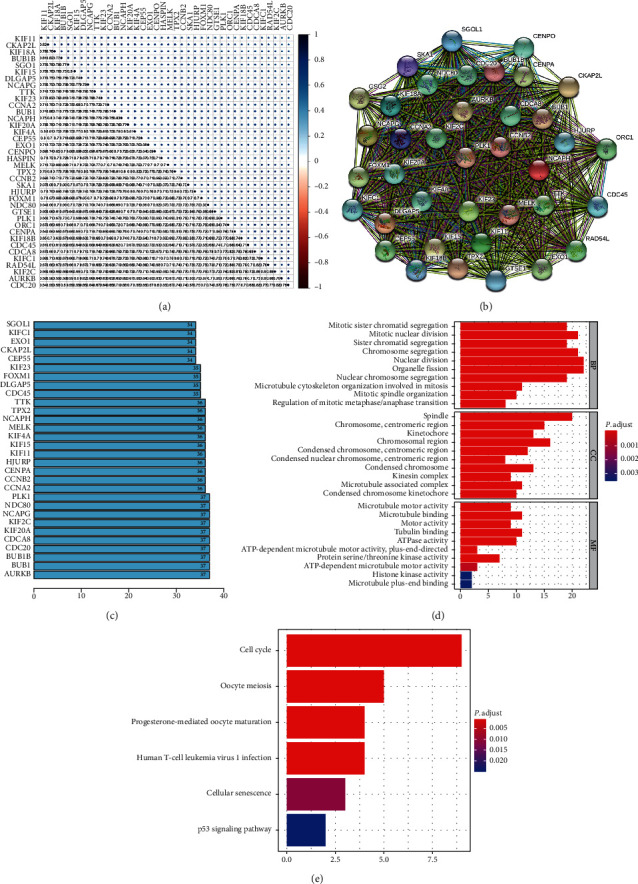
(a) The coexpression of the 38 key genes. The correlation with the least connection was between *CDC20* and *KIF11* (0.54), whereas the highest connection was between *BUB1* and *CKAP2L* (0.86). (b) STRING database to construct the protein-protein interaction network and the number of adjacent nodes of each gene showed by a histogram. (c) GO analysis results of key genes in the turquoise module. (d, e) KEGG analysis results of key genes in the turquoise module.

**Figure 4:**
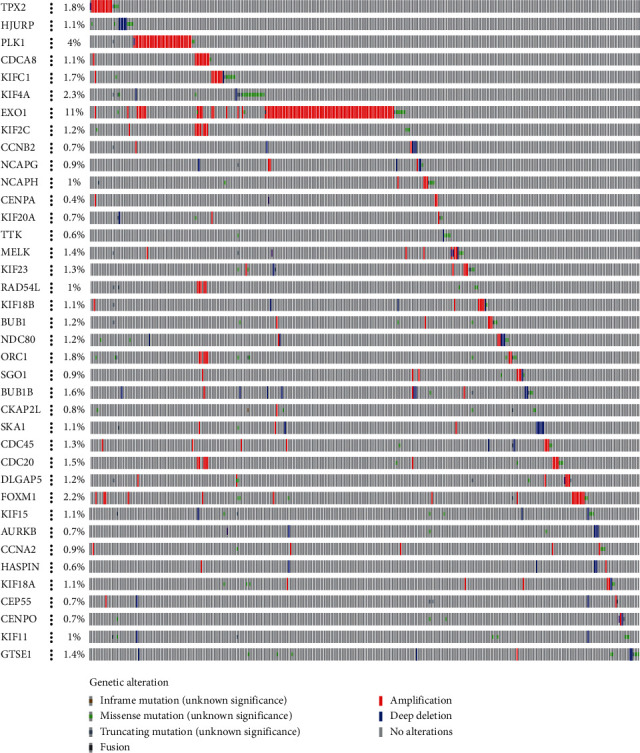
Key genes were modified in 869 of 2173 (40%) BC patients, and amplification was the most common type of modification in BC.

The different expression transcriptional levels of these 38 key genes in cancers and normal samples were first analyzed; all key genes were significantly highly expressed in BC tissues (Figure 1(c)), and the heatmap of these 38 key genes was shown (Figure 1(d)). The coexpression of these 38 key genes was investigated next. The least was between *CDC20* and *KIF11* (0.54), whereas that with the highest connection was between *BUB1* and *CKAP2L* (0.86) (Figure 3(a)). The analysis of the interactions and effects within these 38 genes provides convincing information about the mechanisms and development of the disease. Therefore, the STRING database was used to construct the protein-protein interaction network (Figure 3(b)), and the number of adjacent nodes of each gene is displayed in a histogram (Figure 3(c)). The most adjacent node genes were *PLK1, NDC80, NCAPG, KIF2C, KIF20A, CDCA8, CDC20, BUB1B, BUB1*, and *AURKB*, indicating that these genes may be the hub genes in the network. Next, cBioPortal showed that these 38 key genes were modified in 869 of 2173 (40%) BC patients, and amplification was the most common modification type in BC (Figure 4).

### 3.4. Functional Annotation of Turquoise Modules

Gene enrichment was carried out using the “clusterProfiler” R software package to illuminate the functional similarity of the module genes. The results showed that changes in BP of stemness-related genes were significantly enriched in mitotic sister chromatid segregation, mitotic nuclear division, chromosome segregation, organelle fission, nuclear division, and nuclear chromosome segregation. Changes in MF were mainly enriched in microtubule motor activity, microtubule binding, motor activity, tubulin binding, ATPase activity, and protein serine/threonine kinase activity. Changes in CC of DEGs were mainly enriched in the spindle, chromosome, centromeric region, kinetochore, chromosomal region, condensed chromosome, and condensed chromosome, centromeric region (Figure 3(d)). The KEGG pathway analysis showed that the DEGs were mainly enriched in the cell cycle, oocyte meiosis, progesterone-mediated oocyte maturation, human T-cell leukemia virus 1 infection, cellular senescence, and p53 signaling pathway (Figure 3(e)).

### 3.5. Prognosis Value of Key Genes in BC

**Figure 5:**
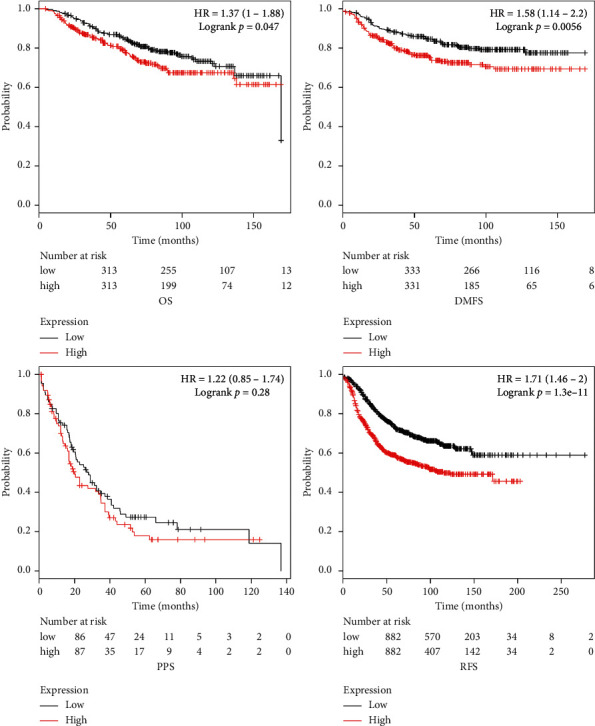
Prognosis value of key genes including OS (overall survival), PPS (postprogression survival), DMFS (distant metastasis-free survival), and RFS (relapse-free survival).

The publicly available Kaplan–Meier plotter datasets were used to determine what role key genes play in BC patients. The Kaplan–Meier curve and log-rank test analyses (Figure 5) indicate that lower expression of key genes was also significantly associated with longer overall survival (OS) (HR = 1.37, 95% CI: 1–1.88, *P*=0.047), distant metastasis-free survival (DMFS) (HR = 1.58, 95% CI: 1.14–2.2, *P*=0.0056), and relapse-free survival (RFS) (HR = 1.71, 95% CI: 1.46–2, *P*=1.3*e* − 11). These results suggest that key genes play an important role in cancer patients' prognosis.

### 3.6. Immune Infiltrates Correlation with the Most Adjacent Node Genes in BC

**Figure 6:**
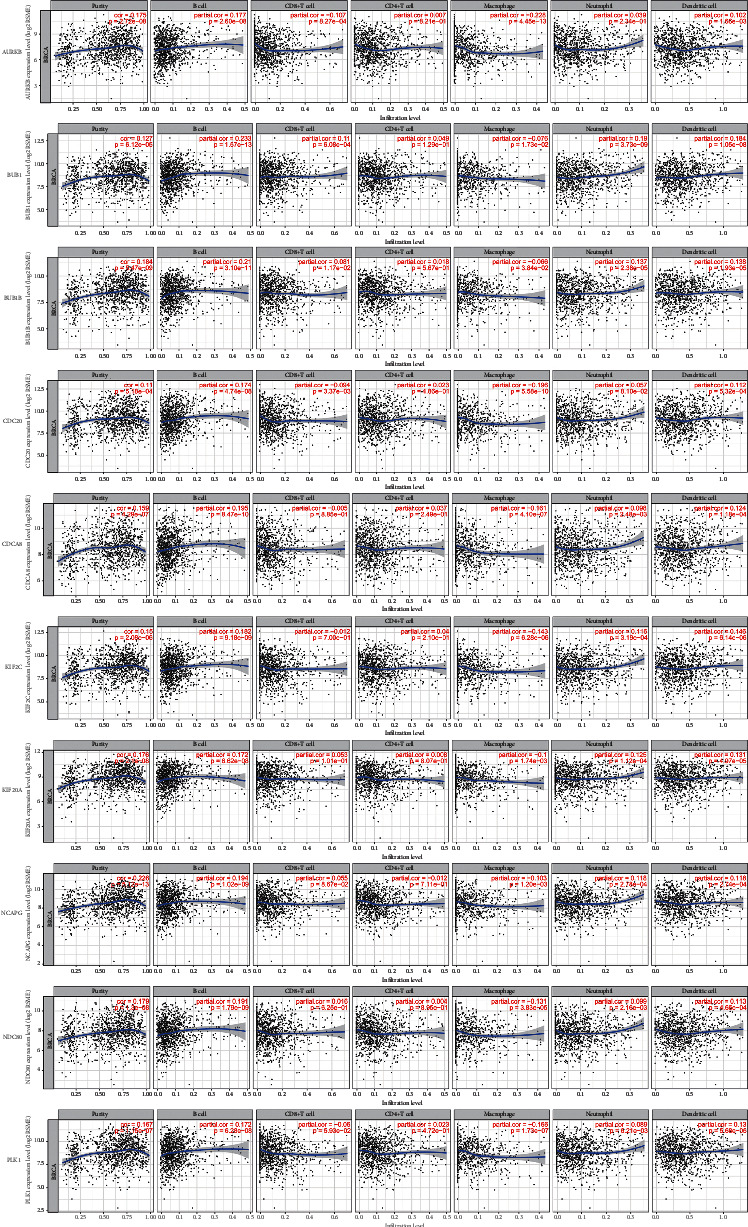
Immune infiltration based on six kinds of immune cells (B cells, CD8^+^ T cells, CD4^+^ T cells, macrophages, neutrophils, and dendritic cells) of hub genes.

This study had obtained adjacent node genes prior. The associations between immune infiltrates and these node genes were interactively explored using the Timer database which provides 6 major analytic modules. The results indicated that partial genes are connected with certain immune cells, positively as well as negatively; these correlations do not seem very extreme. The specific relationship between immune infiltration and key genes needs further exploration (Figure 6).

## 4. Discussion

This study conducted a comprehensive and detailed assessment of key genes associated with CSC characteristics by integrating WGCNA with the corrected mRNAsi of BC, based on Pan et al. statistics. Moreover, the associations of these genes with clinicopathologic characteristics, function, immune infiltrates, and expression differences were explored. CSCs play a vital role in tumor progression, therapeutic resistance, and recurrence and thus may provide a new kind of targeted therapy. Breast cancer has high morbidity and mortality. We attempt to identify upstream genes for early identification and early treatment of BC. This may attract much attention in the diagnosis of BC and may uncover potential biomarkers or targets as determinants for prognosis.

All organs and tissues develop from pluripotent stem cells. Recent evidence suggests that strategies that induce differentiation of CSCs could make a difference in the eradication of tumor cells, which means suppressing certain transcription factors could affect tumor recurrence [21]. Breast cancer stem cell pools are associated with many elements, of which lipid metabolism cannot be ignored [22]. Typical BC therapies include surgery, radiation, and chemotherapy. However, stem cells maintain extremely high reproduction and migration rates; therapies that do not target stem cells are linked with higher tumor recurrence rates [22]. This study sheds new light on screening potential upstream targeting genes. The stem cell-like characteristics and loss of the differentiated phenotype are a display of cancer progression; there was an extremely higher expression level in tumor samples compared to normal ones. Our results demonstrated that mRNAsi correlates significantly with the patient T2 and 4 and Stage 2 and 4 classification, suggesting that stem cell properties rise in the middle and large phases of cancer course.

The key genes were selected from the turquoise module rooted at GS and MM. The coexpression within these modules of the connection with the least correlation was between *CDC20* and *KIF11* (0.54), whereas that with the highest connection was between *BUB1* and *CKAP2L* (0.86). Furthermore, from the histogram map, the most adjacent node genes which may be considered as point keys in the network were *PLK1, NDC80, NCAPG, KIF2C*, *KIF20A, CDCA8, CDC20, BUB1B, BUB1*, and *AURKB*. Through Oncomine, it was found that genes were all upregulated, of which *NCAPG* and *KIF20A* were particularly clear. Chen et al. found that non-SMC condensin I complex subunit G (*NCAPG*) may be the key genes of TNBC [23]. Gong et al. showed that the overexpression of *NCAPG* could promote HCC cell proliferation and reduce HCC cell apoptosis [24]. Studies have reported that Cdc20 is a pivotal mitotic factor governing anaphase initiation, and Cdc20-APC/C is fast becoming highlighted as a key instrument in tumor progression [25]. Evidence has shown that *CDC20*, a significant cell division regulator, exhibits an oncogenic function and plays vital roles in tumorigenesis and progression of solid tumors [26]. We hypothesize that *NCAPG* and *CDC20* are potential drug therapeutic targets, needing further exploration.

The cBioPortal was used to explore modification species analysis of stemness-related DEGs; 38 key genes were found to be modified in 869 of 2173 (40%) BC patients, and amplification is the most common type of modification in BC. The functions and pathways of 20 stemness-related genes were analyzed; GO and KEGG showed that changes in KEGG were significantly enriched in the cell cycle, oocyte meiosis, progesterone-mediated oocyte maturation, human T-cell leukemia virus 1 infection, cellular senescence, and p53 signaling pathway. Our study highlights a key gene *CDC20*, which is recognized to be closely connected with the cell cycle [25]. Immunotherapy is an emerging epidemic, so we also investigated the immune infiltrate correlation with key genes; the results showed they were extremely connected with immune cells. Zhang et al. found that a negative immune regulator interleukin-1 receptor type 2 (IL1R2) is upregulated in breast cancer (BC) tissues, especially in breast tumor-initiating cells (BTICs) [27].

In conclusion, key genes were found to play indispensable roles in BC stem cell maintenance, which agrees with previous studies. The important role of stemness-related genes in the prognosis of BC was also demonstrated. However, there are limitations to our study. (1) Clinical trials are needed in the future to confirm these results and promote the application of these key genes in prognosis evaluation. (2) The conclusions are based on retrospective data, and more research is required to certificate these findings.

## Data Availability

The datasets analyzed in the current study are available in the TCGA repository (http://cancergenome.nih.gov/).

## References

[1] Urooj T., Wasim B., Mushtaq S., Shah S. N. N., Shah M. (2020). Cancer cell-derived secretory factors in breast cancer associated lung metastasis: their mechanism and its future prospects. *Current Cancer Drug Targets*.

[2] Desantis C. E., Ma J., Goding Sauer A., Newman L. A., Jemal A. (2017). Breast cancer statistics, 2017, racial disparity in mortality by state. *CA: A Cancer Journal for Clinicians*.

[3] Mallika S. D., Kondapalli K., Amos S. J., Venkanteshan P. (2014). Breast cancer statistics and markers. *Journal of Cancer Research and Therapeutics*.

[4] Rainey L., Eriksson M., Trinh T. (2020). The impact of alcohol consumption and physical activity on breast cancer: the role of breast cancer risk. *International Journal of Cancer*.

[5] Oh J., Yoon H.-J., Jang J.-H., Kim D.-H., Surh Y.-J. (2019). The standardized Korean Red Ginseng extract and its ingredient ginsenoside Rg3 inhibit manifestation of breast cancer stem cell-like properties through modulation of self-renewal signaling. *Journal of Ginseng Research*.

[6] Prat A., Pineda E., Adamo B. (2015). Clinical implications of the intrinsic molecular subtypes of breast cancer. *The Breast*.

[7] Zamora A., Alves M., Chollet C. (2019). Paclitaxel induces lymphatic endothelial cells autophagy to promote metastasis. *Cell Death & Disease*.

[8] Hao N., Shen W., Du R. (2019). Phosphodiesterase 3A represents a therapeutic target that drives stem cell-like property and metastasis in breast cancer. *Molecular Cancer Therapeutics*.

[9] Sand A., Piacsek M., Donohoe D. L. (2020). WEE1 inhibitor, AZD1775, overcomes trastuzumab resistance by targeting cancer stem-like properties in HER2-positive breast cancer. *Cancer Letters*.

[10] Zaoui M., Morel M., Ferrand N. (2019). Breast-associated adipocytes secretome induce fatty acid uptake and invasiveness in breast cancer cells via CD36 independently of body mass index, menopausal status and mammary density. *Cancers*.

[11] Gašparović Č., Milković L., Dandachi N. (2019). Chronic oxidative stress promotes molecular changes associated with epithelial mesenchymal transition, NRF2, and breast cancer stem cell phenotype. *Antioxidants (Basel)*.

[12] Pan S., Zhan Y., Chen X., Wu B., Liu B. (2019). Identification of biomarkers for controlling cancer stem cell characteristics in bladder cancer by network analysis of transcriptome data stemness indices. *Frontiers in Oncology*.

[13] Li J., Liu C., Chen Y. (2019). Tumor characterization in breast cancer identifies immune-relevant gene signatures associated with prognosis. *Frontiers in Genetics*.

[14] Liu J., Chen Y., Gao C. (2019). Tumor characterization in breast cancer identifies immune-relevant gene signatures associated with prognosis. *Frontiers in Genetics*.

[15] Niemira M., Collin F., Szalkowska A. (2019). Molecular signature of subtypes of non-small-cell lung cancer by large-scale transcriptional profiling: identification of key modules and genes by weighted gene co-expression network analysis (WGCNA). *Cancers*.

[16] Altermann E., Klaenhammer T. R. (2005). PathwayVoyager: pathway mapping using the Kyoto Encyclopedia of genes and genomes (KEGG) database. *BMC Genomics*.

[17] Szklarczyk D., Franceschini A., Wyder S. (2015). STRING v10: protein-protein interaction networks, integrated over the tree of life. *Nucleic Acids Research*.

[18] Wu P., Heins Z. J., Muller J. T. (2019). Integration and analysis of CPTAC proteomics data in the context of cancer genomics in the cBioPortal. *Molecular & Cellular Proteomics*.

[19] Li D., Zhong J., Zhang G., Lin L., Liu Z. (2019). Oncogenic role and prognostic value of MicroRNA-937-3p in patients with breast cancer. *OncoTargets and Therapy*.

[20] Li T., Fan J., Wang B. (2017). TIMER: a web server for comprehensive analysis of tumor-infiltrating immune cells. *Cancer Research*.

[21] Rahmati M., Johari B., Kadivar M., Rismani E., Mortazavi Y. (2020). Suppressing the metastatic properties of the breast cancer cells using STAT3 decoy oligodeoxynucleotides: a promising approach for eradication of cancer cells by differentiation therapy. *Journal of Cellular Physiology*.

[22] Hershey B. J., Vazzana R., Joppi D. L., Havas K. M. (2019). Lipid droplets define a sub-population of breast cancer stem cells. *Journal of Clinical Medicine*.

[23] Chen J., Qian X., He Y., Han X., Pan Y. (2019). Novel key genes in triple-negative breast cancer identified by weighted gene co-expression network analysis. *Journal of Cellular Biochemistry*.

[24] Gong C., Ai J., Fan Y. (2019). NCAPG promotes the proliferation of hepatocellular carcinoma through PI3K/AKT signaling. *OncoTargets and Therapy*.

[25] Chi J., Li H., Zhou Z. (2019). A novel strategy to block mitotic progression for targeted therapy. *EBioMedicine*.

[26] Alfarsi L. H., Ansari R. E., Craze M. L. (2019). CDC20 expression in oestrogen receptor positive breast cancer predicts poor prognosis and lack of response to endocrine therapy. *Breast Cancer Research and Treatment*.

[27] Zhang L., Qiang J., Yang X. (2019). IL1R2 blockade suppresses breast tumorigenesis and progression by impairing USP15-dependent BMI1 stability. *Advanced Science*.

